# Remarks on the Case of Inflammation of the Abdominal Viscera

**Published:** 1814-08

**Authors:** G. Axbridge


THE
### Medical and Physical Journal.

2 OF VOL. XXXII.] AUGUST, 1814. [NO. 186.

"For many fortunate discoveries in medicine, and for the detection of nume-
"rous errors, the world is indebted to the rapid circulation of Monthly
"Journals; and there never existed any work to which the faculty in
"EUROPE and AMERICA were under deeper obligations than to the
"Medical and Physical Journal of London, now forming a long, but an
"invaluable series."—RUSH.

*For the Medical and Physical Journal.*

### *Remarks on the Case of Inflammation of the Abdominal Viscera;* by Mr. AXBRIDGE.

A CORRESPONDENT has communicated a case to the Public, through the medium of the Medical and Physical Journal for May, which does not excite astonishment by any peculiarity of symptoms, so much as by a strange peculiarity of treatment; so strange, indeed, that I am surprised he should have published the case, after having by dissection so decidedly convinced himself of the nature of the complaint, and his error in the treatment.

This gentleman describes the case to be "Inflammation of the Abdominal Viscera, principally in the Kidneys and Bladder, occasioned by excessive Debauchery;" and his patient's symptoms, when he first saw him, were clearly those of high inflammation of some of the abdominal viscera; "hot skin, and exquisite sensibility of the surface of the belly, particularly at the pit of the stomach; acute pains in the right kidney, inability to pass urine, bowels costive;" his indications were, "the warm bath, emollient enemas, and a bladder of *cold* water to the stomach."—Trusting to the efficience of these, he suffers his patient to remain "upwards of five days," the symptoms daily increasing in danger: "bilious vomiting, sleepless nights, yellow skin, eyes sunk, and hollow, loaded tongue, pains in the loins most acute, obstinate constipation, and total retention of urine; pulse frequent, small, and hard, with such extreme sensibility of the abdomen, that pressure made for the ordinary purpose of examination could not be borne;" then, and not till then, he acknowledges that the "violence of the symptoms left him in no hopes of a recovery." Yet something more must be done: he therefore says, "*emollient drinks* and *enemas* were now prescribed, with *cordial* and *antispasmodic* draughts.' This undoubtedly produced a change in the symptoms; for we find the patient "*expectorating*, instead of vomiting, bilious matter;" and in a few hours he dies.

Mr. Jones may have his reasons for publishing this case; for my part, I would most carefully have concealed it within the pages of my day-book, knowing, although we are not in possession of any certain remedy for that *singular* disease "*Ecchymosis of the Heart*;" yet a mere novice would have tried plentiful bleeding, purging, blistering, &c. &c. for acute inflammation of the abdominal viscera, rather than have risked the chance of losing his patient, by trusting so confidently as he did to the effects of "emollient drinks and emollient enemas."*

*June* 18, 1814. G. AXBRIDGE.

---

*For the Medical and Physical Journal.*

* We cannot admit the correctness of the principle that would induce our ingenious correspondent to withhold an important fact in pathology on account of errors in practice; an extensive collection of such would be a most important acquisition to medical literature, would serve as a beacon to warn the practitioner of his danger, and in this respect would be as useful to him as a chart of rocks and shoals is to the mariner. In our estimation, to confess, or what is the same in effect to relate, an error, indicates an ingenuousness of disposition highly commendable, and certainly not deserving of censure. We may remark also, though we agree generally with the author of this paper on the point of practice, that one part of it is less objectionable than he seems to imagine—the application of cold to the parietes of the abdomen in cases of inflammation of its viscera, we know to have been attended with marked advantage. In a recent case of *puerperal peritonitis*, it acted like a charm, and produced a perfect remission of the pain, which the most copious detraction of blood had been scarcely able to mitigate for an hour. ED.

